# Selenium protects against LPS-induced MC3T3-E1 cells apoptosis through modulation of microRNA-155 and PI3K/Akt signaling pathways

**DOI:** 10.1590/1678-4685-GMB-2019-0153

**Published:** 2020-06-08

**Authors:** Yan Huang, Zhen Jia, YongQiang Xu, MeiLan Qin, SiYin Feng

**Affiliations:** 1 Department of Orthopaedics, Hunan Provincial People's Hospital, Changsha, Hunan Province, P.R. China.

**Keywords:** Selenium, apoptosis, miR-155, PI3K/AKT, LPS

## Abstract

Bone infection or osteomyelitis is usually a complication of inflammation-related traumatic bone injury. Selenium has been shown to have potential cytoprotective effects and the ability to reduce oxidative stress and apoptotic events in osteomyelitis, but the exact mechanism remains unclear. Here, we used LPS-induced apoptotic MC3T3-E1 cells and aimed to confirm selenium's protective effect on cell apoptosis as well as to investigate the underlying mechanisms of this role. Our investigation confirmed selenium-mediated inhibition of LPS-induced cell apoptosis and ROS accumulation in MC3T3-E1 cells. Upon selenium treatment, the bcl-2 levels were upregulated, while the levels of Bax and cyto-C were down-regulated. Furthermore, these effects were accompanied by the suppression of miR-155 and the phosphorylation of protein kinase B (Akt). A more in-depth study demonstrated that LY294002 (a specific inhibitor of PI3K), abolished the selenium-mediated cytoprotective effect of MC3T3-E1 cells against LPS-induced injury and down-regulation of miR-155. In general, these results demonstrated that selenium exerts a cytoprotective effect by attenuating cell apoptosis and oxidative damage via a PI3K/Akt/miR-155-dependent mechanism.

## Introduction

Osteomyelitis is an infection of bones, typically characterized by bone destruction leading to bone resorption and dysfunction (Ian, 2013). It can affect both children and adults, and usually develops in the limbs, especially the hip joints, the distal femur and proximal tibia, but any bone can be infected (Hatzenbuehler and Pulling, 2011). Although osteomyelitis can be caused by aerobic or anaerobic bacteria and mycobacteria, a major pathogen causing the infection is *Staphylococcus aureus*, which is a gram-negative bacteria, and account for approximately 37-80% of all the incidences of osteomyelitis (Wright and Nair, 2010; Kalinka *et al.*, 2014). LPS, an important cell wall component of gram-negative bacteria, a virulence factor that has been demonstrated to induce cell apoptosis *in vitro* (Guo *et al.*, 2014). Previous studies found that LPS injection induced low turnover osteopenia and enhanced osteoblast apoptosis in TNFR1-/- mice (Ochi *et al.*, 2010). Thus, the main objective of this study was to identify potential drugs that can inhibit LPS-mediated apoptosis in mouse osteoblast cell line.

MicroRNAs (miRNAs) are small non-coding RNAs that act as post-transcriptional regulators of gene expression either by causing the degradation of target mRNAs or inhibition of their translation (Vrabec *et al.*, 2018). MiRNAs have been demonstrated to play critical roles in cell apoptosis, proliferation, differentiation and invasion, contributing to the pathogenesis of many diseases (Vojtechova *et al.*, 2018). A previous study found that miR-155 plays essential roles in inflammatory responses and auto-immune diseases (Kuo *et al.*, 2013). A recent study found that silencing of miR-155 ameliorated inflammation in a systemic lupus erythematosus rat model (Xin *et al.*, 2015). Similarly, other studies found that in synovial mononuclear cells, overexpression of miR-155 lead to enhanced expression of proinflammatory factors (Jin *et al.*, 2014) and that gene knockdown of miR-155 can reduce LPS inflammatory damage to in microglia cells (Yin *et al.*, 2017). However, these studies did not explore the roles and mechanisms of miR-155 in inflammation seen in osteomyelitis. In our study, we explored the effects of miR-155 on MC3T3-E1 cells viability, apoptosis and the underlying pathways.

Selenium (Se) is one of the essential trace elements, which regulates cellular redox homeostasis as part of immune regulation and acts as an antioxidant in humans and animals (Diwakar *et al.*, 2017). Several studies have extensively characterized the selenium-dependent functions in humans and clarified its physiological and pathophysiological effects (Benstoem *et al.*, 2015; Dinh *et al.*, 2018). Selenium has been shown to confer a protective effect on oxidative stress and apoptosis via the mitochondrial pathway (Pastaci Özsobaci *et al.*, 2018). Other data suggest a correlation between selenium and cellular inflammatory responses, especially the inflammatory response induced by LPS (Wang *et al.*, 2018). However, the role of selenium in LPS-induced apoptosis of osteoblast cell line is still unclear. In this study, we explored whether selenium protects MC3T3-E1 cells against LPS-induced apoptosis and investigated the related pathways that selenium acts on. We first examined the cytoprotective effect of selenium against LPS-mediated MC3T3-E1 cells injury. Furthermore, we examined the level of miR-155 and the activation of PI3K/Akt pathway in this process.

## Material and Methods

### Reagents

In this study, sodium selenite was obtained from Sigma Aldrich (St. Louis, MO, USA). We treated the cells with 4 ng/ml of sodium selenite (Zhu *et al.*, 2011). LPS (E.coli O111:B4), MTT, paraformaldehyde, Triton X-100, DMSO and trypsin were purchased from Sigma Aldrich. We obtained DMEM, FBS and penicillin/streptomycin from Gibco Inc (Rockville, MD, USA). The Annexin V/FITC Apoptosis Detection Kit I was obtained from Clontech (Clontech Laboratories Inc, USA). The ECK kit was purchased from Pierce Inc. Antibodies to Bax, Bcl-2, p-AKT, TRITC-conjugated goat anti-mouse IgG and β-actin were obtained from Santa Cruz Inc.

### Cell culture

In this study, the murine pre-osteoblast cell line, MC3T3-E1 was purchased from Shanghai Cell Bank. MC3T3-E1 cells were grown in DMEM medium containing 10% FBS, with penicillin (100 U/mL) and streptomycin (100 U/mL). MC3T3-E1 cells were grown in a humidified incubator (95% air and 5% CO_2_ at 37 °C).

### MTT assay

Cytotoxicity exerted by LPS on MC3T3-E1 cell line was assessed by MTT assay. Briefly, MC3T3-E1 cells were seeded (5000 cells/well) in a 96-well plate in triplicates, incubated for 24 h, and treated with (0-800 ng/ml) LPS for different hours (24, 48 and 72 h) in 96-well plates. In order to examine the protective effect of selenium, cells were treated with different concentrations of LPS (0-800 ng/ml) in the presence or absence of 4 ng/ml sodium selenite for 24 h. After incubation, 10 μl of MTT (5 mg/mL) reagent was added to each well and incubated for another 4 h in dark with 5% CO_2_ at 37 °C. The cell supernatants were removed and 150 μl DMSO was added to each well. After 5 min, we detected the absorbance at 490 nm using a microplate reader (Thermo Fisher Scientific, Inc., Pittsburgh, PA, USA). The experiments were performed in triplicate. Percentage of viable cells in each treatment concentrations were calculated as a ratio of sample OD to the control OD.

### Cell transfection

The miR-155 inhibitor (5'-GTGTAACACGTCTAT ACGCCCA-3') and the corresponding negative control (inhibitor NC, 5'-GTGTAACACGTCTATACGCCCA-3') were from GenePharma (Shanghai, China). Lipofectamine 3000 (Thermo Fisher Scientific, Waltham, MA, USA) was used to implement transient transfection as the manufacturer's instruction. Briefly, MC3T3-E1 cells were harvested by trypsinization and then resuspended in 6-well plates at a concentration of 5×10^5^/well. Cells were incubated in DMEM with 10% FBS at 37 °C until confluence reached 60%, then cultured in DMEM containing transfection working solution (500 μl/well) for 4 h. Subsequently, the cells were replaced with new medium for 24-48 h at 37 °C with 5% CO_2_, for the following experiment.

### Analysis of apoptosis

To differentiate the surviving and apoptotic cells, flow cytometry analysis was performed to measured and quantify apoptosis by the Annexin V-FITC/PI apoptosis detection kit. Cells were plated into 6 wells (3 × 10^5^ cells/mL). After the appropriate treatments, cells were harvested by 0.25% trypsin and washed with PBS and then suspended in 300 μl 1×binding buffer. Thereafter, the solution for cell apoptosis detection Annexin V was added in the dark-for 15 min indoor temperature. The cells were centrifuged at 1500 rpm for 5 min and the supernatant was discarded. Re-suspending with buffer solution was performed. Finally, 10 μl propidium iodide (PI) was added. Another 400 μl of binding buffer was added, and the cells were analyzed using a FACScan (Becton Dickinson, NY, USA). Annexin V-FITC and PI emissions were detected in the FL 1 and FL 2 channels. (AnnexinV-FITC)-/PI+, the cells in this area are necrotic cells. (AnnexinV+FITC)+/PI+, the cells in this region are late apoptotic cells. (AnnexinVFITC)+/PI-, the cells in this region are early apoptotic cells. The percentages of normal, early apoptotic, late apoptotic, and necrotic cells were calculated using CellQuestTM software (Becton–Dickinson).

### TUNEL

Apoptosis was also determined using a TUNEL method (Roche Applied Science, Penzberg, Germany) according to the manufacturer's instructions. Firstly, the pretreated cells were fixed in 4% PFA for 1 h room temperature. Subsequently, we fixed the cells in 2:1 v/v ethanol/acetic acid for 10 min room temperature. Next, TUNEL reaction mixture (containing 5 μl enzyme solution and 45 μl fluorochrome-labeled solution) was added to the cells, which were cultured for 60 min at 37 °C in the dark. Finally, MC3T3-E1 cells were incubated with DAPI for 15 min. The cells were analyzed using a fluorescence microscope.

### Detection of intracellular ROS level

ROS generation was detected by FACS analysis according to the manufacturer's instructions. In brief, MC3T3-E1 cells were stained with 5 μg/ml 2',7'-dichlorodihydrofluorescein diacetate (DCF-DA) for 30 min at 37 °C. Then the cells were washed and subjected to flow cytometry using Becton-Dickinson FACS Caliber.

### Quantitative Real-Time PCR

For miR-155 detection, the cDNA was synthesized using the miRNA cDNA Synthesis Kit (GeneCopoeia, Wuhan, China). The qRT-PCR was performed using SYBR Green Master Mix (Yeasen Biotechnology). U6 was used as internal reference. The expression of miR-155 was quantified using the 2-ΔΔCt relative quantification method.

The primers for the target genes were as follows: miR-155: 5'-GCAGCTAGCCCAGGGTTGGAACTGAG TTTGA-3'; 5'-GCAAAGCTTCAGTTAACCCGGCGGT GA-3'.U6:5'-CTCGCTTCGGCAGCACA-3';5'-AACGC TTCACGAATTTGCGT-3'. The size of miRNA fragments is 250 bp.

### Immunofluorescence staining

After washing twice with 1 × PBS, we fixed the MC3T3-E1 cells with 4% paraformaldehyde for 60 min at 4 °C. Next, the cells were permeabilized with TritonX-100 for 15 min and incubated with p-AKT antibody (1:400) overninght. The next morning, MC3T3-E1 cells were incubated with TRITC-conjugated goat anti-mouse IgG (1:100) for 30 min in dark. Then, Hoechst33342 was added for 15 min. At last, we used fluorescence microscopy to get images.

### Preparation of proteins in the mitochondrial and cytosolic fractions and Western blot analysis

Whole-cell lysates were prepared using ice-cold extract buffer [20 mM Western blotting analysis Hepes–KOH,1 mM EDTA,1.5 mM MgCl2, 1 mM EGTA,1 mM DTT, and 0.1 mM phenylmethanesulfonyl fluoride (PMSF)]. The resuspended cells were homogenized and centrifuged twice at 750 × *g* for 10 min at 4 °C. The supernatants were centrifuged at 10,000 × *g* for 15 min at 4 °C to obtain the mitochondrial pellets. Cytosolic fractions were obtained after further centrifugation at 100,000 × *g* for 1 h at 4 °C. The protein samples were quantified by BCA Protein Assay Kit. Equal amounts of proteins (20 µg) were separated by 10% SDS-PAGE gels, and then transferred to PVDF membranes, which were blocked for 2 h with 5% non-fat milk before incubated with primary antibodies: Bax(1:400), cyto-C(1:400),p-AKT(1:400) and β-actin (1:1000) overnight at 4 °C. The membranes were incubated with HRP-conjugated secondary antibody (Santa Cruz Bio-technology) for 2 h. Finally, the protein bands were visualized using an enhanced chemiluminescence reagent (Pierce).

### Statistical analysis

All data was analyzed using SPSS18.0 software and expressed as the mean ± SEM. Significances were analyzed with One way ANOVA and Tukey's multiple comparison tests. *P<0.05, **P<0.01 were considered statistically significant.

## Results

### LPS induces apoptosis in MC3T3-E1 cells

**Figure 1:**
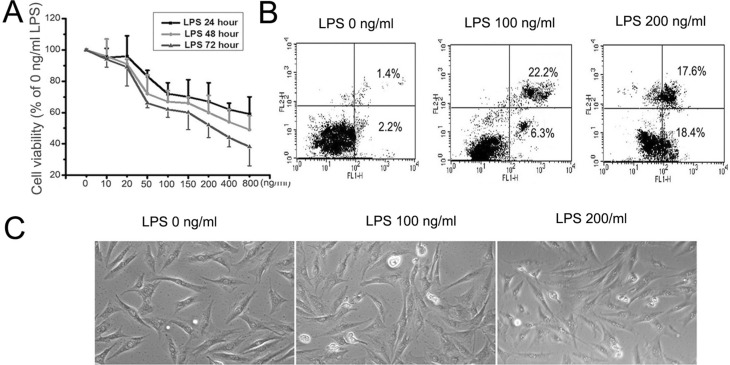
The effect of LPS on cell apoptosis in MC3T3-E1 cells. (A) MC3T3-E1 cells were treated with 0-800 ng/ml LPS for 24, 48 and 72 h, and cell viability was detected by MTT. The data were mean ± SEM (n=5). (B) MC3T3-E1 cells were treated with 0 ng/ml, 100 ng/ml 200 ng/ml LPS for 24 h, and the apoptosis was detected with annexin V-FITC/PI double staining. Results are representative of three experiments. (C) Cells were treated as aboved and we observed the morphology of the cells under light microscope.

To examine cell viability after LPS treatment, 0-800 ng/ml LPS was added to the medium for various times (24, 48, and 72 h), and the cell viability was assessed by MTT. The data revealed that LPS decreased cell viability in a time and dose-dependent manner (Figure 1A). At both 100 and 200 ng/ml of LPS, the cell viability showed significant decline (P<0.01). Therefore, 100 and 200 ng/ml concentrations were selected as experimental concentrations for use in subsequent experiments. As shown in Figure 1B, compared to the control group, the LPS groups (100, 200 ng/ml of LPS) showed markedly elevated apoptotic rates. Culturing MC3T3-E1 cells with 100 ng/ml of LPS enhanced their apoptosis to 28.5%, while culturing the cells with 200 ng/ml of LPS enhanced cell apoptosis to 36%. Consistently, similar results were observed by inverted microscopy (Figure 1C). After treatment with LPS, we looked for cell population with condensed or fragmented nuclei under the microscope, and found cells expressing the markers of early and late apoptosis. These findings suggested that LPS could promote MC3T3-E1 cells to undergo apoptosis *in vitro*.

### Selenium attenuated LPS-induced cell damage

**Figure 2:**
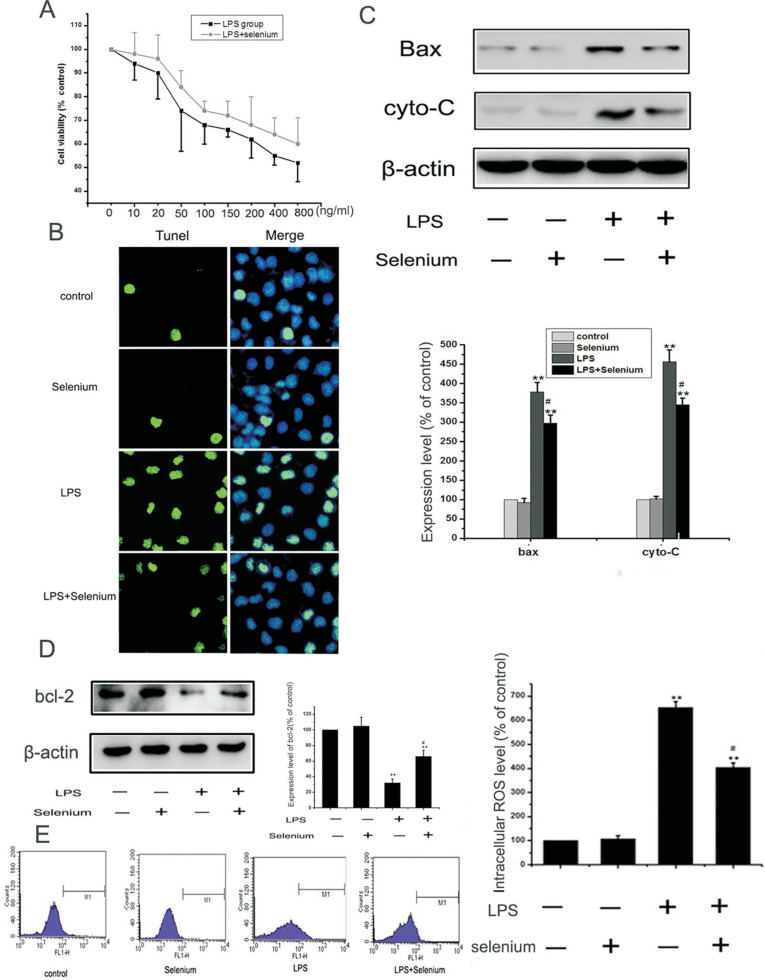
Selenium inhibited LPS-induced apoptosis and oxidative stress in MC3T3-E1 cells. (A) We incubated MC3T3-E1 cells with 0-800 ng/ml of LPS with or without 4 ng/ml sodium selenite for 48 h, and cell viability was detected by MTT. The data were mean ± SEM (n=5). (B) MC3T3-E1 cells were incubated in 200 ng/ml LPS in the presence or absence of 4 ng/ml sodium selenite for 24 h, and the apoptosis was assessed by using TUNEL analysis. (C,D) Cells were treated as above, western blots were performed with the antibodies indicated. Relative expression of Bax, cytochrome c and bcl-2 were calculated and normalized to the loading control β-actin. The data were mean ± SEM (n = 6). (** P < 0.01, vs. control, #P< 0.05, LPS vs. LPS+selenium group). (E): Cells were treated as aboved, and the level of intracellular ROS production were detected by FACS analysis. The data were mean ± SEM (n = 3). (** P < 0.01, vs. control, #P< 0.05, LPS vs. LPS+selenium group).

To examine if selenium could protect MC3T3-E1 cells from cell injury by regulating apoptosis, we subjected the LPS-stimulated MC3T3-E1 cells to selenium. Firstly, we assessed the cell viability by MTT after 24-hour long culture with 0-800 ng/ml LPS to the medium with or without selenium. As shown in Figure 2A, selenium apparently increased the cell viability compared to LPS alone. Furthermore, TUNEL staining demonstrated the protective effects of selenium on LPS-induced apoptosis of MC3T3-E1 cells. In the LPS plus group, there was an apparent decrease in cell shrinkage and nuclear condensation compared to LPS alone group (Figure 2B). We also found that LPS significantly elevated apoptosis-related proteins including bax and cyto-C in western-blot experiments. In contrast, addition of selenium showed a remarkable decline in the levels of these proteins (Figure 2C). In the LPS group, the level of the anti-apoptotic protein, bcl-2, was significantly reduced, while in LPS plus selenium group, bcl-2 protein was significantly elevated (Figure 2D). These data suggested that selenium treatment notably ameliorated LPS-induced cell apoptosis.

Many studies have found that ROS produced by the mitochondria, could induce oxidative stress, promote apoptosis, and lead to cell death (Aintzane *et al.*, 2012). To investigate whether selenium could reduce LPS-induced ROS production, we assessed the intracellular ROS levels by FACS analysis. As shown in Figure 2E, the level of ROS was elevated upon LPS stimulation. However, in the presence of selenium, the ROS levels showed a notable decline. In summary, these results indicated that LPS-induced cell injury is significantly inhibited by selenium treatment.

### LPS induced cell injury by upregulatting the level of miR-155

**Figure 3:**
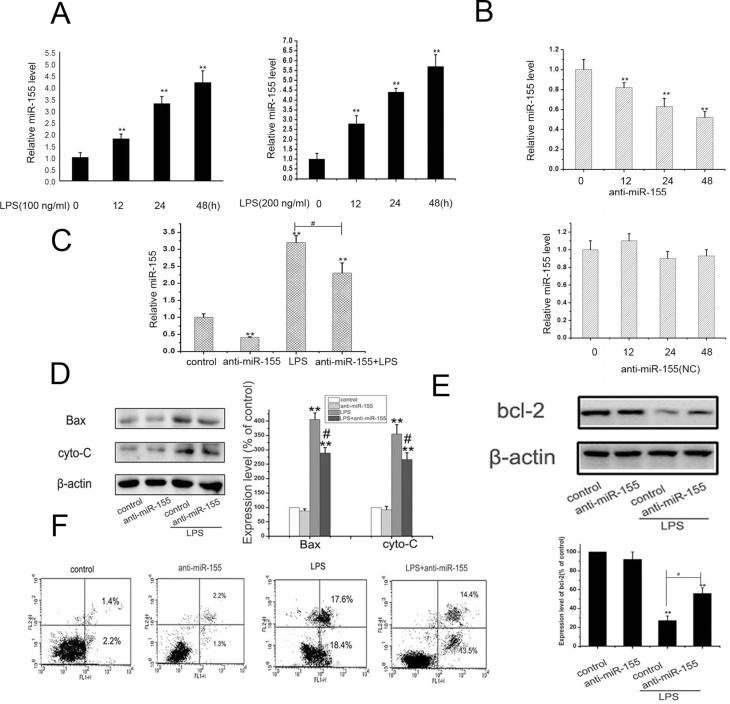
Knock-down of miR-155 inhibited LPS-induced cell apoptosis. (A) MC3T3-E1 cells were cultured with 100,200 ng/ml LPS for 0, 12, 24 and 48 h, and the level of miR-155 was detected by qRT-PCR, U6 was as the loading control. The data were mean ± SEM (n=5). (** P<0.01, vs. 0 h group). (B): miR-155 inhibitor and inhibitor control (NC) were transfected using Lipofectamine 3000 and cultured for 0, 12, 24 and 48 h and the level of miR-155 was detected by qRT-PCR, U6 was as the loading control. The data were mean ± SEM (n=6). (** P<0.01, vs. 0 h group). (C): MC3T3-E1 cells were transfected with miR-155 inhibitors and cultured for 24 h. Then, the control cells and anti-miR-155 cells (after transfection for 48 h) were cultured with or without 200 ng/ml LPS treatment for 24 h and the level of miR-155 mRNA was detected by qRT-PCR. U6 was as the loading control. The data were mean ± SEM (n=6). (** P <0.01, vs. control group; # p<0.01 LPS group vs. LPS+anti-miR-155 group). (D,E) Cells were treated as aboved, western blots were performed with the antibodies indicated. Relative expression of Bax, cytochrome c and bcl-2 were calculated and normalized to the loading control β-actin. The data were mean ± SEM (n = 6). (** P < 0.01, vs. control, # P < 0.05, LPS vs. LPS+anti-miR-155 group). (F) Induction of apoptosis in LPS-induced cells was detected with annexin V-FITC/PI double staining. Results are representative of three experiments.

Previous studies have shown that miR-155 plays an important role in inflammatory responses and autoimmune diseases (Sun *et al.*, 2019). Other studies have found that in mice, inhibition of miR-155 could alleviate autoimmune inflammatiory response (Zhang *et al.*, 2018). However, the role of the effect of miR-155 in bone infection is still unclear. So, we next planned to explore the functional effect of miR-155 in MC3T3-E1 cell injury induced by LPS. Upon LPS stimulation, we first measured the expression of miR-155 in MC3T3-E1 cells by qRT-PCR. As shown in Figure 3A, the level of miR-155 was significantly upregulated in a time-dependent manner upon LPS stimulation compared to the control (P<0.01). These results suggest that miR-155 might play an important role in LPS induced cell damage. Next, we investigated whether the expression of miR-155 would be affected by LPS stimulation in MC3T3-E1 cells. Thus, we transfected miR-155 inhibitor or control inhibitor (NC) into MC3T3-E1 cells to alter the miR-155 mRNA level, and explored the role of miR-155. We found the best inhibition rate after 48 hours of transfection (Figure 3B). In order to examine the effect of miR-155, we cultured cells LPS in presence or absence of miR-155 inhibitor, and determined the level of miR-155 by qRT-PCR. As shown in Figure 3C, LPS significantly increased miR-155 expression, whereas anti-miR-155 significantly decreased the miR-155 level.

To verify the role of miR-155 on LPS-induced cell injury, we verified cell apoptosis by western blot. The results showed that LPS increased Bax and cyto-c protein levels and decreased the bcl-2 levels. Knock-down of miR-155 reduced the upregulation of Bax and cyto-c and increased the down-regulation of bcl-2 induced by LPS (Figure 3 D, E). Flow cytometry results (Figure 3F) showed that LPS increased apoptosis and increased the level of miR-155 in MC3T3-E1 cells (P<0.01), while the down-regulation of miR-155 could inhibit MC3T3-E1 cells apoptosis (P<0.01). 200 ng/ml of LPS markedly enhanced apoptosis in MC3T3-E1 cells to 36%, while anti-miR-155 treatment led to a drop of apoptosis rate to 27.9%. Transfection of miR-155 inhibitors not only reduced the level of miR-155 but also rescued MC3T3-E1 cells from apoptosis. These findings suggested that miR-155 inhibition protected MC3T3-E1 cells against LPS-induced cytotoxicity.

### Selenium down-regulated the level of miR-155.

**Figure 4:**
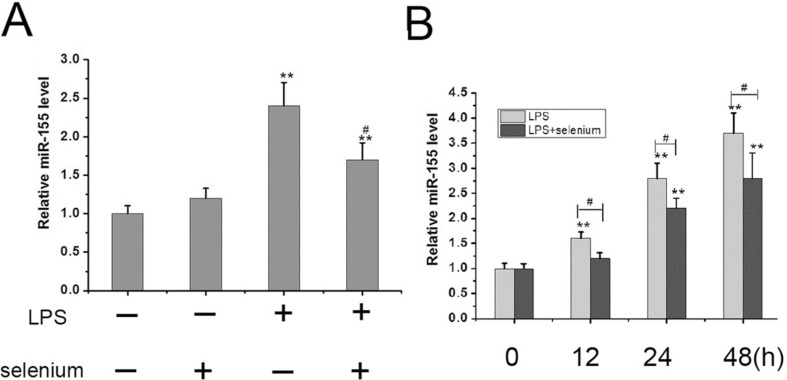
Selenium decreased the level of miR155 in MC3T3-E1 cells. (A) MC3T3-E1 cells were incubated in 100 ng/ml LPS in the presence or absence of 4 ng/ml sodium selenite for 24 h, and the level of miR-155 was detected by qRT-PCR. The data were mean ± SEM (n=6). (** P < 0.01, vs.control;# P < 0.05, LPS vs. LPS+selenium group). (B) MC3T3-E1 cells were treated with 200 ng/ml LPS in the presence or absence of 4 ng/ml sodium selenite for 0, 12, 24 and 48h,and the level of miR-155 was detected by qRT-PCR. The data were mean ± SEM (n=6). (** P < 0.01, vs.0 hour group;# P < 0.05, LPS vs. LPS+selenium group).

Our study has revealed that miR-155 plays an important role in apoptosis and in LPS-induced inflammatory response in MC3T3-E1 cells. To explore whether selenium could control the level of miR-155 in MC3T3-E1 cells, we performed qRT-PCR on the cells following LPS treatment with or without selenium. We found that the level of miR-155 was increased by LPS compared to the control. However, the combination of LPS and selenium significantly reduced the level the miR-155 (P <0.01, Figure 4A). We next examined whether selenium treatment would led to decrease the up-regulation of miR-155 induced by LPS. We pre-treated MC3T3-E1 cells with selenium and monitored miR-155 levels over time. We found that the level of miR-155 levels in the LPS plus selenium group were lower than those in the LPS alone group at different culture times (12, 24, 48h) (Figure 4B). Overall, the data demonstrate that selenium protected cells from apoptosis via down-regulation of miR-155.

### Selenium protected cells through PI3K/AKT/miR-155 signaling pathway

**Figure 5:**
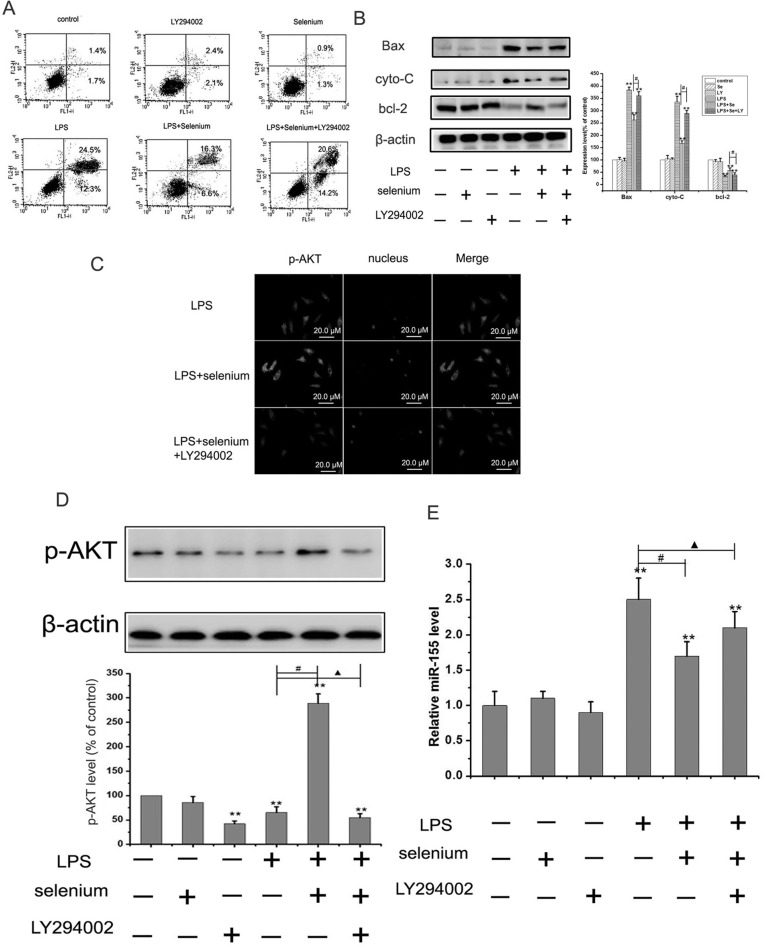
Selenium inhibited cells apoptosis and regulated miR-155 level through PI3K/Akt signaling pathway. (A) MC3T3-E1 cells were incubated in 100 ng/ml LPS in the presence or absence of 4 ng/ml sodium selenite with or without 10 μM LY294002 for 24 h, and the apoptosis was detected with annexin V-FITC/PI double staining. Results are representative of three experiments. (B) Cells were treated as aboved, western blots were performed with the antibodies indicated. Relative expression of Bax, cytochrome c and bcl-2 were calculated and normalized to the loading control β-actin. The data were mean ± SEM (n = 6). (** P < 0.01, vs. control, #P< 0.05, LPS +Se vs.LPS +Se+LY group). (C): Cells were treated as above, and the p-AKT protein was stained and observed under a fluorescence microscope. (D): Cells were treated as above, and the p-AKT protein was detected by western blot. The data were mean ± SEM (n=6). (** P < 0.01, vs.control group;# P < 0.05, LPS vs. LPS+selenium group; ▲P>0.05 LPS vs. LPS+selenium+LY294002 group). (E) Cells were treated as aboved, and the miR-155 was detected by qRT-PCR. The data were mean ± SEM (n = 6). (** P < 0.01, vs.control group;# P < 0.05, LPS vs. LPS+selenium group; ▲P>0.05 LPS vs. LPS+selenium+LY294002 group).

The PI3K/AKT signaling pathway contributes to cell fate decisions by regulating many types of apoptosis (Sulaiman *et al.*, 2019). Based on these findings, we hypothesized that selenium could protect cells from apoptosis by regulating the PI3K/AKT pathway. To examine this, we incubated MC3T3-E1 cells with 20 μM of LY294002 (the inhibitor of PI3K/AKT) (Li *et al.*, 2019). We found that selenium rescued the cells from LPS-induced apoptosis. However, when the cells were pretreated with LY294002, the protective effect of selenium disappeared (Figure 5A). Notably, western-blot results (Figure 5B) verified these findings. The protective effect of selenium disappeared under the action of LY294002. We next measured whether selenium could induce the phosphorylation of Akt by western blotting. Our study found that selenium treatment led to an apparent increased in the level of p-AKT protein. However, co-treatment of MC3T3-E1 cells with selenium and LY294002 effectively inhibited the elevation of p-Akt levels (Figure 5 C, D). This data suggests that selenium could inhibit LPS-induced upregulation of miR-155.

Lastly, we explored whether selenium treatment changed the expression of miR-155 in MC3T3-E1 cells through the PI3K/AKT pathway. As shown in Figure 5E, selenium decreased upregulation of miR-155 in MC3T3-E1 cells. However, upon pre-treatment with LY294002, the effect of selenium disappeared. Overall, the data indicated that selenium protected MC3T3-E1 cells from LPS-induced injury by attenuating apoptosis and down-regulating the expression of miR-155 via PI3K/AKT signaling pathway.

## Discussion

Osteomyelitis is a common and frequently occurring disease in orthopedics affecting bone remodeling and resulting in massive destruction of bone tissue. *Staphylococcus aureus* infection accounts for a large proportion of the disease causality. However, effective therapies for bacteria-associated bone damage is limited. Lipopolysaccharides (LPS), a major component of gram-negative bacterial membranes has been shown to cause inflammatory osteolysis, including osteomyelitis, implants infection, and septic arthritis (Mörmann *et al.*, 2008). Therefore, in this study, we decided to study the underlying mechanisms of LPS-induced osteoblasts damage, and explored therapeutic options to protect against these damages. In our study, we used LPS to induce apoptosis of MC3T3-E1 cells and explore the protective of selenium on MC3T3-E1 cell apoptosis.

Selenium is a trace element important to maintain physiological functioning and health of the human body. In recent years, the benefits of selenium to human health have been gradually recognized. Selenium deficiency often leads to various disorders, such as Keshan disease, diabetes and thyroid dysfunction (Brozmanová *et al.*, 2010; Duntas *et al.*, 2015). Studies have demonstrated anti-apoptosis activity of selenium both *in vitro* as well as *in vivo* (Brozmanová *et al.*, 2010; Yüksel *et al.*, 2017; Defo Deeh *et al.*, 2019). Thus, in this study we examined the effect of selenium on osteoblasts apoptosis. We found that LPS could significantly inhibit MC3T3-E1 cells viability, promote apoptosis and increase ROS production *in vitro*.

Apoptosis, also known as programmed cell death, has also been found to be associated with oxidative stress and proinflammatory cytokines (Hu *et al.*, 2007). Previous study found that selenium could scavenge free radicals *in vitro* and inhibit the growth of microorganisms (Huang *et al.*, 2003; Tran *et al.*, 2011). Our research confirmed this finding. We found that selenium reversed LPS-mediated increase in Bax and cytochrome-c expression and decreased the level of the miR-155 (Figure 2). Consistent with previous findings, our study demonstrated that inhibition of miR-155 dramatically increased cell viability and reduced cell apoptosis in LPS-injured MC3T3-E1 cells (Figure 3). Further analyses demonstrated that miR-155 knockdown could lead to a decrease in miR-155 expression, which in turn protected MC3T3-E1 cells against LPS-induced injury. Additionally, our study showed that selenium inhibited miR-155 expression directly (Figure 4). Our study further found that while LPS promoted cell damage by upregulating the level of miR-155 in MC3T3-E1 cells, selenium protected the cells from the LPS-induced injury via down-regulation of miR-155.

Several studies have extensively demonstrated that the PI3K/Akt signaling is an important pathway involved in preventing MC3T3-E1 against oxidative stress and apoptosis (Jin *et al.*, 2017; Wu *et al.*, 2018). Therefore, we hypothesized that the cytoprotective effect of selenium against LPS-induced apoptosis in MC3T3-E1 cells could be related to activation of PI3K/Akt signaling pathways. As expected, we found that selenium treatment increased the levels of phosphorylated Akt compared with the LPS group. Notably, the use of the PI3K inhibitor LY294002 demonstrated pharmacological inhibition of PI3K which in turn inhibited selenium-mediated protection against LPS injury, as evident by a decrease in cell viability. Therefore, we concluded that PI3K/Akt signaling may play an important role in the protective effects of selenium. Moreover, our study also found that selenium markedly reduced the expression of the miR-155 level in MC3T3-E1 cells and activated the PI3K/AKT signaling pathway, which was blocked upon culture with PI3K inhibitor LY294002 (Figure 5). Previous study also found that the enhancement of miR-155 expression and/or activity could play a critical role in reversing the effect of PI3K/AKT signaling pathway (Tu *et al.*, 2017). Taken together, these results demonstrated that selenium modulates the PI3K/AKT/miR-155 signaling pathway by suppressing LPS-induced apoptosis.

In conclusion, our study demonstrated that selenium protected against LPS-induced MC3T3-E1 cells apoptosis through modulation of miR-155 and PI3K/Akt signaling pathways. Accordingly, our findings suggest that selenium may be valuable for future treatment of osteomyelitis and furthermore, miR-155 may be a novel molecular therapeutic target for the development of a novel treatment modality for osteomyelitis.
